# Assessing Breast Cancer through Tumor Microenvironment Mapping of Collagen and Other Biomolecule Spectral Fingerprints—A Review

**DOI:** 10.1021/acssensors.4c00585

**Published:** 2024-08-23

**Authors:** Diya Pratish Chohan, Shimul Biswas, Mrunmayee Wankhede, Poornima Menon, Ameera K, Shaik Basha, Jackson Rodrigues, Darshan Chikkanayakanahalli Mukunda, Krishna Kishore Mahato

**Affiliations:** ‡ Manipal School of Life Sciences, Manipal Academy of Higher Education, Karnataka, Manipal 576104, India; § Department of Biophysics, Manipal School of Life Sciences, Manipal Academy of Higher Education, Karnataka, Manipal 576104, India

**Keywords:** Collagen, Breast cancer, Photoacoustic, Tumor microenvironment, Spectra, Absorbance, Mapping, NADH, FAD, Elastin

## Abstract

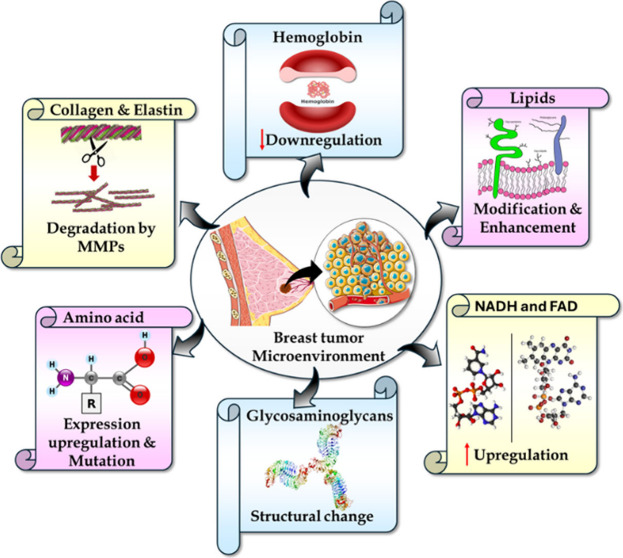

Breast cancer is a major challenge in the field of oncology, with around 2.3 million cases and around 670,000 deaths globally based on the GLOBOCAN 2022 data. Despite having advanced technologies, breast cancer remains the major type of cancer among women. This review highlights various collagen signatures and the role of different collagen types in breast tumor development, progression, and metastasis, along with the use of photoacoustic spectroscopy to offer insights into future cancer diagnostic applications without the need for surgery or other invasive techniques. Through mapping of the tumor microenvironment and spotlighting key components and their absorption wavelengths, we emphasize the need for extensive preclinical and clinical investigations.

Breast cancer is the most prevalent malignancy among women worldwide and persists as a daunting obstacle within the field of oncology. Despite the advancing technologies available that vastly contribute toward efficient diagnostics and therapeutics, the occurrence of breast tumors keeps rising with approximately 2.3 million cases detected and around 670,000 deaths across the globe according to the GLOBOCAN 2022 statistics.^1^ Researchers tend to explore the microenvironment of breast cancer to find novel insights into the molecular details that control tumor development and progression.^2,3^ The extracellular matrix (ECM) appears to be a vital regulator influencing the carcinogenesis processes.^4^ Emerging evidence suggests changes in the tissue components present in the ECM are finely linked to tumorigenic growth, invasion, and metastasis, making it a crucial aspect to be studied as they can reveal vital molecular features present within the tumor microenvironment for future applications.^5−8^

Traditionally, collagen, a major structural protein component found in the ECM has been projected as a fixed scaffold providing structure, support, and strength to tissues.^9−11^ However, recent findings indicate that alterations in the collagen structure dynamically impact the behavior of tumors and their responses to treatment in cases of breast cancer. Understanding this relationship is crucial for developing innovative approaches to disease prognosis, diagnosis, and treatment. Although multiple techniques such as Histopathology, Mammography, Ultrasound Imaging, Computed Tomography (CT), and Magnetic Resonance Imaging (MRI) have been utilized in the process of detection and surveillance of breast cancer, they cannot frequently offer comprehensive insights into collagen dynamics within the cancerous tissue microenvironment.^12−17^ These approaches are challenged due to diagnostic restrictions in dense breast tissues, radiation exposure, time constraints, skill expertise, limited penetration, and costly false positives and false negatives.^18−21^ Introducing photoacoustic spectroscopy, a technique that detects biomolecular signals by leveraging the photoacoustic effect. When a sample is excited with a specific wavelength modulated/pulsed light, the molecules undergo optical absorption and get excited, and move to higher energy states. The excited molecules undergo localized heating, followed by thermoelastic expansion and relaxation. This creates pressure variation generating acoustic signals, which are then detected using suitable acoustic detectors (microphone/PZT), and analyzing these waves reveals the optical absorption characteristics of the biomolecules as illustrated in Figure 1.^22−24^ This method provides very good sensitivity and specificity in identifying relative collagen content differences between cancer subtypes.^25^ Its outstanding sensitivity enables the detection of trace biomolecule concentrations, and its high specificity allows for precise differentiation of unique biomolecular signatures, even within intricate biological settings.^26−28^ This technique also offers real-time visualization of the molecular composition and architecture of biomolecule signatures within breast cancer tissues.^29,30^

**Figure 1:**
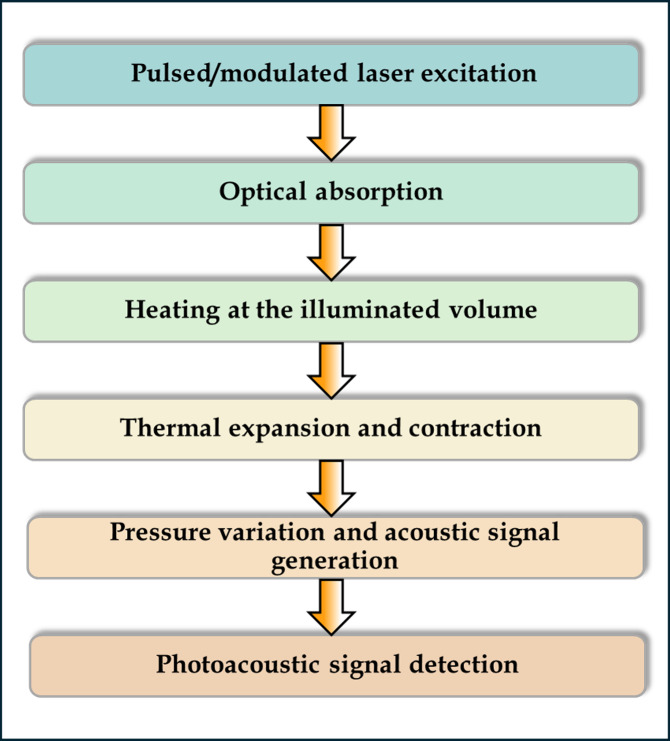
Flowchart illustrating photoacoustic signal generation.

This review explores the complex world of collagen biology and photoacoustic spectroscopy, uncovering how they are connected and impact breast cancer pathogenesis. It delves deeper into the diverse range of 28 collagen types, their classification, and their functional significance toward growth, advancement, invasion, and metastasis of breast tumors (Table 1). It also highlights the potential of photoacoustic collagen signals as vital indicators for detecting and understanding breast cancer by elucidating the absorption ranges and distinctive photoacoustic peaks linked to different collagen types (Table 2). This may serve as a powerful foundation for future applications in mapping tumor microenvironments and personalized medicine. Moreover, this review uncovers the complex composition of breast tumor surroundings, emphasizing the contributions of different components like Elastin, Flavin Adenine Dinucleotide (FAD), Nicotinamide Adenine Dinucleotide Hydride (NADH), Glycosaminoglycans (GAGs), and many others toward carcinogenesis. It also elucidates their spectral wavelengths, opening new paths for biomarker discovery and early diagnostics.

**Table 1:** Role of Various Collagen Types within the Breast Tumor Microenvironment^a^

Collagen type	Collagen family	Gene name	Location in chromosome	Role in breast cancer	Effect on tumorigenesis when upregulated	Reference
I	Fibril-forming	COL1A1	17q21.33	Stimulates Orai 1 channel expression and basal calcium ions influx, regulating breast cancer cell growth, and metastasis and silencing their apoptotic rate.	Positive	(42, 43)
		COL1A2	7q21.3			
II	Fibril-forming	COL2A1	12q13.11	-	-	(43, 44)
III	Fibril-forming	COL3A1	2q32.2	Downregulation of collagen III forms pro-carcinogenic stroma promotes proliferation, adhesion, and metastasis of breast tumor cells, and inhibits apoptosis.	Negative	(43, 45)
IV	Network-forming	COL4A1	13q34	Promotes advancement of luminal breast cancer by enhancing the expression of c-Myc oncogene, which in turn facilitates glycolytic activity in tumor cells.	Positive	(43, 46)
		COL4A	13q34			
		COL4A3	2q36.3			
		COL4A4	2q36.3			
		COL4A5	Xq22.3			
		COL4A6	Xq22.3			
V	Fibril-forming	COL5A1	9q34.3	Leads to specific siRNA-induced breast cancer cell viability, invasion, and migration.	Positive	(43, 47)
		COL5A2	2q32.2			
		COL5A3	19p13.2			
VI	-	COL6A1	21q22.3	Reduced expression of collagen VI leads to loss of tubular structure to solid structure and contributes to mitotic nature and metastasis.	Negative	(43, 48)
		COL6A2	21q22.3			
		COL6A3	2q37.3			
		COL6A4P1	3p25.1			
		COL6A4P2	3q22.1			
		COL6A5	3q22.1			
		COL6A6	3q22.1			
VII	-	COL7A1	3p21.31	-	-	(43)
VIII	Network-forming	COL8A1	3q12.1	Associated with vascular remodeling and angiogenesis process. It combines with integrin α2β1 to stimulate propagation and spread of breast tumor cells.	Positive	(43, 49)
		COL8A2	1p34.3			
IX	FACITs	COL9A1	6q13	-	-	(43)
		COL9A2	1p34.2			
		COL9A3	20q13.33			
X	Network-forming	COL10A1	6q22.1	-	-	(43)
XI	Fibril-forming	COL11A1	1p21.1	Activates pro-survival pathways and alters tumor metabolic phenotype leading to cell migration, invasion, and chemotherapy resistance.	Positive	(43, 50)
		COL11A2	6p21.32			
XII	FACITs	COL12A1	6q13-q14.1	Highly associated with collagen I; upregulates cancer growth at various stages by influencing its fibrillation architecture through elevated matrix stiffness and stromal localization. It also leads to invasive metastasis of cancerous cells.	Positive	(43, 51)
XIII	MACITs	COL13A1	10q22.1	Regulates tumor proliferation, metastasis, enhanced cancer cell stemness, induced anoikis resistance and tumorsphere formation.	Positive	(43, 52)
XIV	FACITs	COL14A1	8q24.12	-	-	(43)
XV	Multiplexin	COL15A1	9q22.33	Loss of this collagen type may affect structural integrity of ECM leading to cell invasion, motility, and metastasis.	No effect	(43, 53)
XVI	FACITs	COL16A1	1p35.2	-	-	(43)
XVII	Transmembrane	COL17A1	10q25.1	Suppresses growth of breast tumor cells by deactivating AKT/mTOR signaling pathway.	Negative	(43, 54)
XVIII	Multiplexin	COL18A1	21q22.3	Alters epidermal growth factor receptor tyrosine kinase (ErbB) signaling and combines with EGFR, HER2, and α6 integrin to stimulate the propagation and spread of cancerous cells autonomously.	Positive	(43, 55)
XIX	FACITs	COL19A1	6q13	It has antitumor and antiangiogenic elements as it releases matricryptins that interact with integrin receptors. This complex hinders the phosphorylation of various signaling pathways.	Negative	(43, 56)
XX	FACITs	COL20A1	20q13.33	Linked to reoccurrence and migration of breast cancer cells.	Positive	(43, 57)
XXI	FACITs	COL21A1	6p12.1	-	-	(43)
XXII	FACITs	COL22A1	8q24.23q24.3	Serves as the primary barrier against breast tumor progression, acting as the initial line of defense prior to basement membrane degradation.	Negative	(43, 58−60)
				Not provided. However, it is seen to interact with integrins influencing tumor advancement through its activity in cell adhesion and ECM interaction.		
XXIII	MACITs	COL23A1	5q35.3	-	-	(43)
XXIV	FACITs	COL24A1	1p22.3	-	-	(43)
XXV	MACITs	COL25A1	4q25	-	-	(43)
XXVI	Fibril-forming	COL26A1	7q22.1	-	-	(43)
XXVII	Fibril-forming	COL27A1	9q32	-	-	(43)
XXVIII	-	COL28A1	7p21.3	-	-	(43)

a ACITs - Membrane-Associated Collagens with Interrupted Triple-helices, FACITs - Fibril Associated Collagens with Interrupted Triple helices.

**Table 2:** Collagen Absorbance and Signals in Photoacoustic Technologies

Collagen source	Absorbance range (nm) used in the study	Collagen absorbance peak (nm)	Collagen signals in photoacoustic technology	Reference
Collagen type -I	1100–1300	1200	This study introduced a novel imaging technique, combining ultrasound (US) and photoacoustic (PA), to assess cervical remodeling by evaluating collagen and water content.	(62)
Isolated from BALB/c Nude mice tell	-	1200, 1550, 1700	Effective PA absorption spectrum and optimal wavelength improve PA imaging sensitivity of collagen-based tissues.	(63)
Isolated from rabbit and human bone	1300–1800	1530	The study proposes a PA technique to determine the collagen content of bones as a biomarker for bone health assessment.	(64)
Prostate tissue of human	1200–1690	-	Collagen, hemoglobin, and lipids change during prostate cancer development. Cancerous tissues have more consistent microstructural distributions than normal tissues, as shown by a higher correlation among the ultrasonic power spectra of these chemical components.	(13)
Human skin tissue	680–970	-	This study identified features that differentiate tumors from normal periocular structures. Prevalent melanin in the skin, hemoglobin in the orbital muscle, and collagen in the tarsal plate are used for discrimination.	(65)
Human cervix tissue	1000–1800	1200, 1520, 1540	PA imaging detects collagen organization differences in the cervix between nonpregnant and cesarean groups, with observable spectral changes reflecting alterations in the collagen network.	(66)
Cartilages from human condyles	500–1300	1185	Visible PA spectral changes associated with collagen correlate with varying cartilage damage degrees, aligning with histology and the gold standard Mankin score.	(46)
Rabbit liver tissue	700–960	-	In this study liver fibrosis group showed more collagen fibers in liver than control group, confirmed by ultrasound elastography and pathological results.	(67)
Rat colon tissues	-	1310	Crohn’s disease causes intestinal strictures due to inflammation, fibrosis (high levels of collagen), or a combination of both. This study validates the feasibility of using PAI to assess molecular components and microscopic architectures of these strictures in animal models.	(68)
Rat myocardial tissue	1200–1800	1310	This study uses spectral density ratio to measure collagen and water in heart tissue. This could lead to a minimally invasive probe for cardiovascular diagnosis and therapy.	(69)
Blood-collagen phantom gels	680–980	-	A new photoacoustic (PA) imaging technique directly captures collagen images, the main component of fibrotic tissue. PA collagen imaging is a major advance in measuring fibrosis, with wide preclinical and clinical impact.	(70)
Human skin tissue	1300–1340	1310	This study found that collagen and lipids in the tumor microenvironment can be used as biomarkers for tumor diversity. PA spectral analysis accurately classified tumors based on these biomarkers, providing a new way to diagnose tumors.	(52)

## Collagen Signatures in Breast Cancer

### Structural Changes and Functional Significance

**Figure 2:**
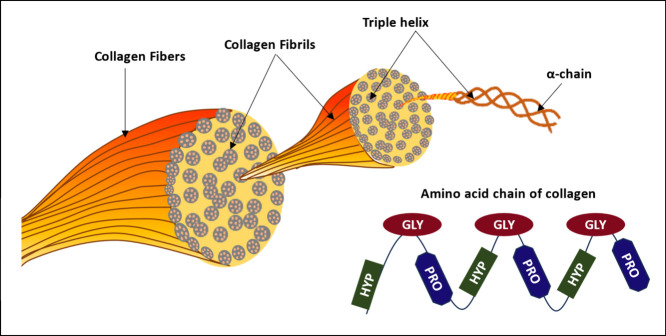
Schematic representation of the collagen structure.

Collagen forms a triple helix structure comprising three polypeptide chains commonly known as alpha chains. These alpha chains may assemble as homotrimeric (same type) or heterotrimeric (different types) configurations based on collagen types, followed by fibril assembly and additional modifications.^31^ The fibrils undergo lateral bonding, forming collagen fibers that align either in an orderly fashion or randomly according to the functional needs of the tissues. The amino acid sequence of collagen consists of repetitive tripeptide units having the sequence Gly-X-Y, where X is proline (Pro), and Y is hydroxyproline (Hyp) as seen in Figure 2.^32,33^ Modifications in these structural configurations contribute to carcinogenesis. The identification of specific Tumor Associated Collagen Signatures (TACS) is gaining prominence as a critical element in both the advancement and the diagnosis of breast cancer. Consequently, they serve as crucial indicators in understanding tumor behavior and its microenvironment, as well as predicting patient prognosis. Some of these collagen structural alterations, together with their functional roles, are covered in this review.

Alterations in the collagen density within breast tissue have a significant role in the advancement of mammary carcinoma. For example, heightened breast density is attributed to the accumulation of collagen I which correlated with a 4–6-fold elevation in the likelihood of breast cancer development.^34^ Additionally, this increased density can result in amplified matrix stiffness, contributing to a 3-fold rise in breast tumor invasion and metastasis.^35^ A study revealed the overdeposition of collagen I, III, and V within the tumor stroma to be a major cause of the development of breast cancers such as Ductal Infiltrating Carcinoma (DIC). This excessive settling of collagen fibers known as desmoplasia creates a dense and fibrotic stroma leading to the formation of a solid lump in breast tissues and evokes neoplastic cell migration.^36^ The degree of straightness and alignment of collagen fibers in breast cancer are highly contrasted in different areas in and around the tumor. Curly, nonparallel collagen fibers were visible in the extratumoral zone, intermediate straightness coupled with a high degree of fiber alignment in the juxta-tumoral zone while being utmost straight within the tumors.^37,38^ This mismatch contributes vastly to the invasive and metastatic behavior of carcinoma cells.^39^ Moreover, the discovery of straight-aligned collagen emerges as an influential independent predictor of unfavorable survival outcomes, whereas cells prefer perpendicular fibers for penetration. Moreover, collagen cross-linking serves as a crucial contributor to the progression, invasion, and spread of breast cancer. The generation of mature collagen fiber cross-links is facilitated by the action of enzymes such as lysyl oxidase (LOX) and lysyl hydroxylases (LH).^40^ This can also lead to robust stiffening and enhanced behavior of the tumors. For example, Hylald-derived collagen cross-linking caused stromal stiffening, tissue fibrosis, and regulated breast tumor aggression in triple-negative (TN) subtype.^41^

Different collagen types can potentially produce distinct photoacoustic effects that could be harnessed for differentiation during detection. This variation may arise from differences in their molecular structures, organization, and interactions with photoacoustic waves. A study distinguished collagen types by their second harmonic generation (SHG) signals and fluorescence lifetimes; for instance, collagen type I is known to generate higher SHG intensity and exhibit longer fluorescence lifetimes compared to collagen type III.^61^ However, to the best of our knowledge, no studies have yet confirmed variations in photoacoustic signals among all collagen types.

### Photoacoustic Signals of Collagen in Different Cancer Types

**Figure 3:**
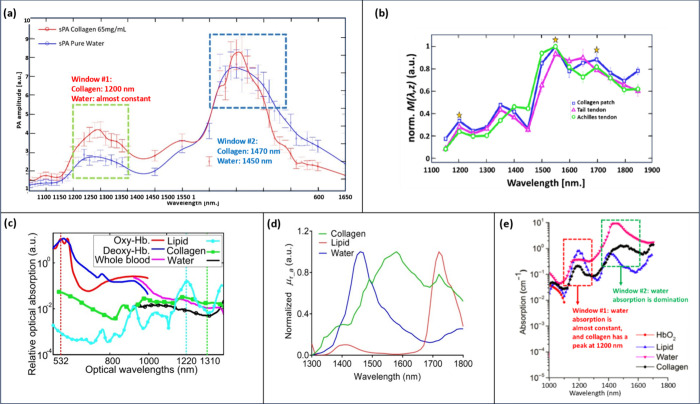
Different studies showing different absorbance peaks of collagen where (a) Collagen has absorption peaks at 1200 and 1470 nm, whereas water is near constant around 1200 nm and has an absorption peak at 1450 nm. (Reproduced from ref (62). Available under the terms of the OSA Open Access Publishing Agreement, Copyright 2019 The Authors.) (b) PA absorption spectrum of collagen appearing at wavelengths of 1200, 1550, and 1700 nm. (Reproduced from ref (63) Available under a Creative Commons Attribution 4.0 Unported License, Copyright 2020 The Authors.) (c) Maximum absorbance at 1310 nm. (Reproduced from ref (68). Available under the terms of the OSA Open Access Publishing Agreement, Copyright 2019 The Authors.) (d) Collagen has a strong absorption peak at 1530 nm. (Reprinted with permission from ref (64). Available under CC BY-NC-ND 4.0 license, Copyright 2021 The Authors.) (e) Optical absorptions of oxyhemoglobin, lipid, collagen, and water are displayed, and here collagen has multiple absorbance peaks at 1200, 1520, and 1540 nm. (Reproduced from ref (66). Available under CC0 1.0 license, Copyright 2021 The Authors.)

Photoacoustic spectroscopy (PAS) offers a distinct approach for analyzing the molecular makeup of tissues, including collagen, in various cancer types. By sensing the absorbance of laser-triggered light pulses, PAS yields valuable insights into the arrangement and dispersion of collagen within tumors. This information is pivotal for comprehending tumor advancement, dissemination, and treatment response. This article delineates the absorbance range and unique collagen peaks observed across various studies of different cancer types (Table 2) and their unique spectra are illustrated in Figure 3.

Table 2 showcases the proficiency of PA spectroscopy in distinguishing tissues with varying collagen content, which is further illustrated in Figure 3, presenting collagen spectra derived from pure collagen solutions and highly collagenous tissues, such as tendons. PA spectroscopy also excels in differentiating collagen from other cellular and extracellular components in complex samples like tumors. Collagen’s unique optical absorption properties enable it to be distinctly identified from other biomolecules. By adjusting the wavelength of the excitation light (for example, 680 to 1,100 nm); PA spectroscopy can specifically target collagen’s absorption peaks, making it stand out among other components. Additionally, advanced spectral analysis techniques (i.e., multispectral PA imaging) can be employed to decompose mixed signals into their individual components based on their unique spectral signals, further enhancing the differentiation process.^64,66,67,71^

## Tumor Microenvironment Mapping

Apart from collagen, numerous other elements inhabit the microenvironment of breast tumors. This review also spotlights these additional components, their respective signal alterations, which may serve as a biomarker for various breast cancer applications, and their analysis using photoacoustic spectroscopy.

### Elastin

**Figure 4:**
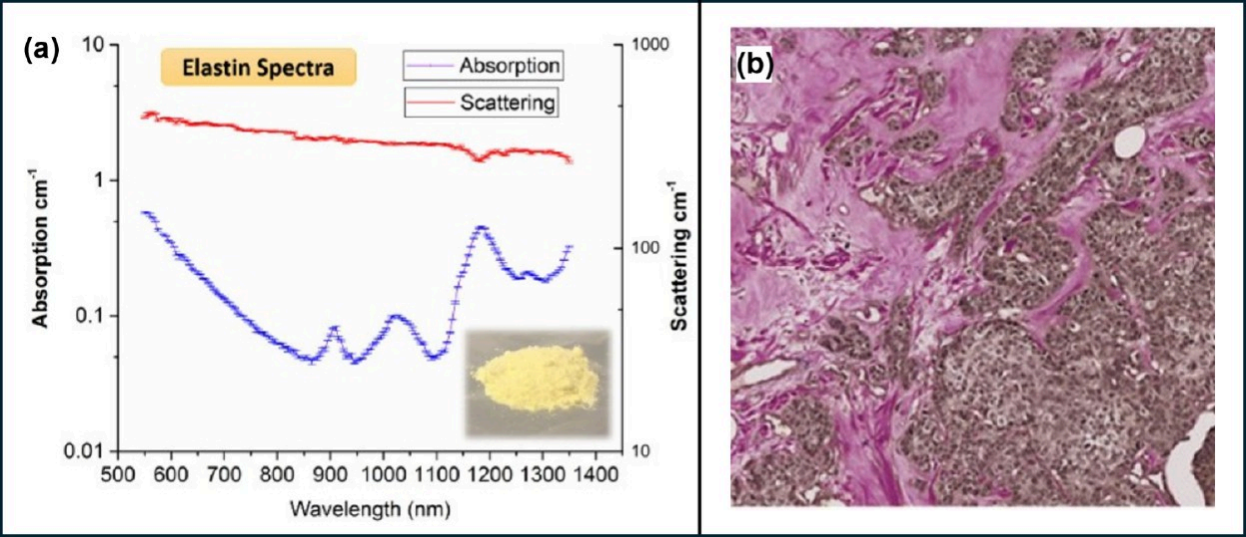
(a) Absorption and reduced scattering spectra of elastin (with standard deviation bars) in blue and red, respectively. (Reprinted with permission from ref (75). Copyright 2017, Elsevier). (b) Representation of elastosis in breast cancer. (Reprinted from ref (76). Available under CC BY 4.0 license, Copyright 2020 The Authors.)

Elastin, often described as a “stretchy” protein is found in the connective tissues throughout the body, providing resilience and flexibility.^72^ Unlike its behavior in healthy tissues, elastin exhibits modifications that affect tissue elasticity and structure which eventually contributes to tumor development, invasion, and metastasis.^6,73^ A recent investigation reported elastosis (bulky aggregates of elastin fibers) to be highly associated with stiffness and malignant breast lesions (Figure 4b).^74^ Research has reported out of 4 peaks (910, 1025, 1185, 1275 nm) in the elastin absorption spectrum measured at 550–1350 nm; the peak at 1275 nm stands out exclusively for elastin (Presented in Figure 4a), making it an optimal marker to differentiate elastin from collagen in tumor microenvironment.^75^

### Nicotinamide Adenine Dinucleotide Hydride (NADH)

NADH is a critical molecule involved in cellular energy production and metabolism. It also serves as a coenzyme in various biochemical reactions i.e. ATP (adenosine triphosphate) generation.^77^ A study unveiled tumor cells produced elevated amounts of reduced forms of NAD+, NADH, and NADPH; inducing mitochondrial dysfunction which increased ROS (reactive oxygen species) formation and damaged mitochondrial DNA (mtDNA); mutations in mtDNA attributed to altered NAD+/NADH ratio which in turn lead to excessive growth of breast cancer cells and metastasis.^78−81^ NADH levels are often upregulated due to heightened metabolic demands and reliance on glycolysis; even in the presence of oxygen (Warburg effect).^82^ Distinct NADH emission spectral bands were found at 439–475 nm when excited at pulsed 266 nm light,^83^ 575 nm when Resorufin dye was used,^78^ and 740–840 nm at 720 nm excitation.^84^

### Flavin Adenine Dinucleotide (FAD)

FAD essentially serves as a coenzyme in several cellular oxidation/reduction metabolic reactions.^85,86^ A change in FAD’s structure critically shifts its biological activity as seen in cancerous cells, contributing to breast tumor survival and proliferation.^87^ For instance, higher FAD concentrations reflect accelerated metabolic functions, energy demands, and proliferative rate of these cells. In addition, cancerous cells display irregular FAD distribution patterns. An experiment on the impact of SLC25A32 (mitochondrial transporter of FAD) on cell survival portrayed that SLC25A32 is highly elevated across different tumor types leading to amplified mRNA expression levels and diminished patient survival rates.^88^ Unique FAD emission spectral bands were pointed out within 502–548 nm when excited at 266 nm pulsed light.^83^

### Glycosaminoglycans (GAGs)

**Figure 5:**
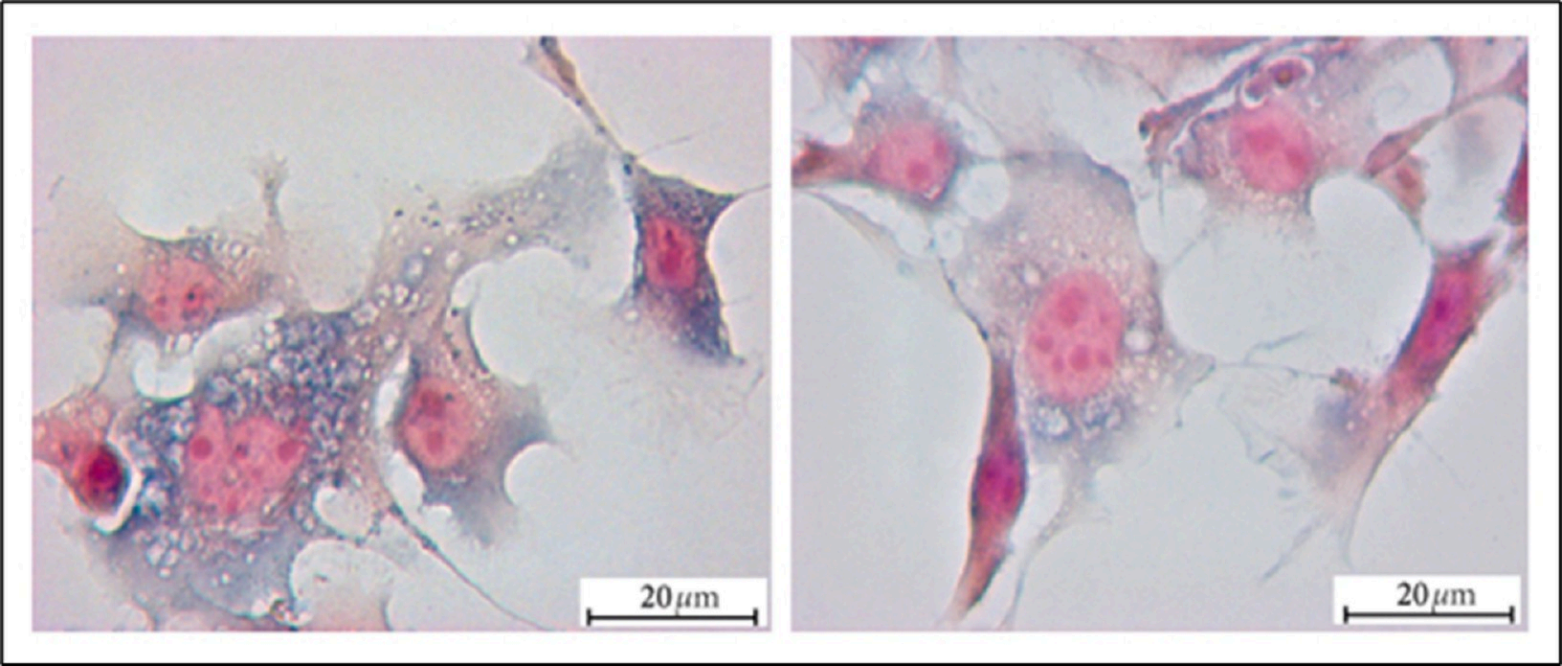
Downregulation of Galectin-3 in breast cancer cells reduces GAG expression. (Reprinted from ref (93). Available under CC BY 4.0 license, Copyright 2019 The Authors.)

GAGs are long, linear polysaccharides found in the ECM of breast tissues which are highly negatively charged in nature.^89−91^ They maintain tissue structure, hydration, and signaling. A study compared healthy breast tissues to breast cancer tissues and displayed differences associated with GAGs such as enhanced sulfation, changes in chain length (15% increase), elevated quantity (2x), and composition.^92^ Pereira et al. indicated that a reduction in Galectin-3 (Gal-3) during breast tumor growth led to modified GAG expression in the cancerous cells (Figure 5). Altered GAG expressions can affect the binding of several ligands, growth factors, and cytokines; modulating their signaling pathways and promoting cancer cell survival, growth, angiogenesis, and metastasis.^93−97^ Glycosaminoglycans exhibit absorption within the NIR-II window, particularly between 1000 and 1800 cm^–1^.^98^ According to our current knowledge of the literature, no studies have recorded photoacoustic spectra exclusively for Glycosaminoglycans (GAGs).

### Matrix Metalloproteinases (MMPs)

**Figure 6:**
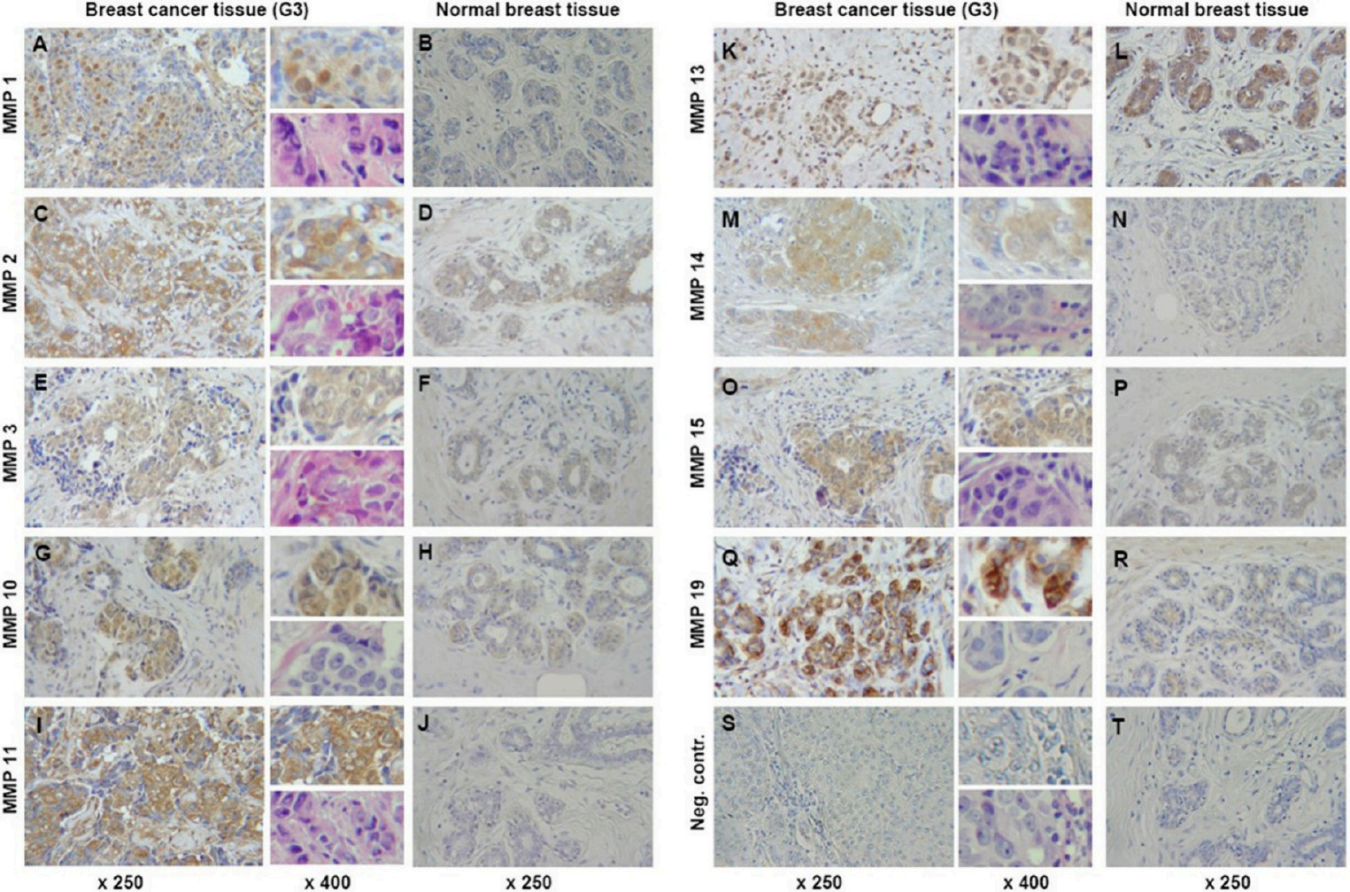
Expression of MMP-1, -2, -3, -10, -11, -13, -14, -15, and -19 (indicated by brown staining) in normal breast tissue and grade 3 (G3) breast cancer tissue. Magnification: ×250 for all images, with detailed views at ×400 in the upper right corner adjacent to the corresponding tumor tissue images; ×400 for HE-staining in the bottom right corner adjacent to the corresponding tumor tissue images. (Reprinted from ref (108). Available under CC BY 2.0 license, Copyright 2009 The Authors.)

These enzymes aid in the breakdown of ECM components, which is vital for a multitude of physiological functions.^99−101^ The degradation of ECM facilitates the generation of new blood vessels around cancerous cells for nutrient supply.^7,102^ MMPs are frequently dysregulated in breast cancer tissues and can modulate the activity of growth factors, cytokines, and cell adhesion molecules; influencing tumor cell behavior and microenvironment.^103,104^ Studies have also linked the levels of MMP-1, -2, -3, -10, -11, -13, -14, -15, and -19 types in the plasma to the stage of breast cancer, inhibition of which can prevent tumorigenesis, invasion, angiogenesis, and migration (Figure 6) illustrates differential expression of MMPs in normal and cancer breast tissue.^4,105−108^

### Hemoglobin (Hb)

**Figure 7:**
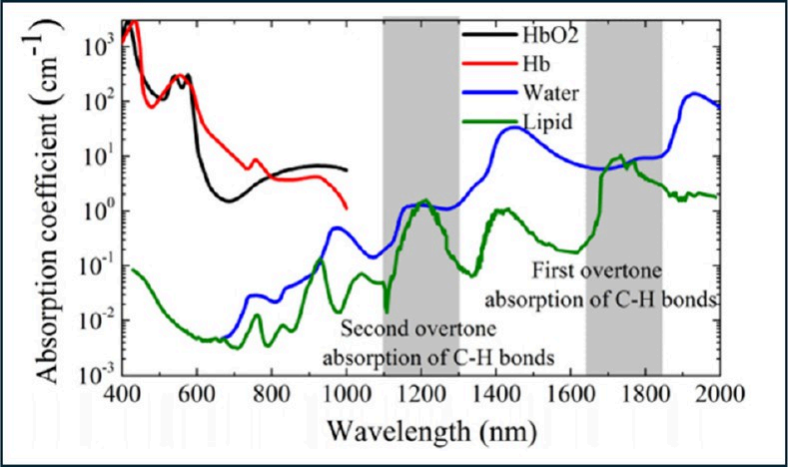
Optical absorption spectra of oxygenated hemoglobin (HbO2), deoxygenated hemoglobin (Hb), water, and lipid for photoacoustic imaging. (Reprinted from ref (117). Available under CC BY 4.0 license, Copyright 2016 The Authors.)

Hb is a protein that ensures vital oxygen (O_2_) delivery to tissues and organs in the body. They are abundantly found in red blood cells and maintain normal physiological function and cellular metabolism.^109^ The level and concentration of Hb vary in the breast carcinoma microenvironment. Research findings have indicated an inverse relationship between breast cancer advancement and Hb levels, alongside direct associations between ferritin levels and disease development.^110,111^ Poor oxygenation status due to decrease Hb content induces regions of hypoxia (low O_2_ levels) which further evokes angiogenesis, cell growth, treatment resistance and metastasis.^112−114^ One study discussed the determination of Hb concentration through PAS by focusing on γ, β, and α peaks in optical absorption spectrum, found at 412, 550, and 580 nm, respectively (Figure 7).^115^ Another study specified absorption variation between highest oxy- and deoxy-Hb at 680 nm while minimal at 808 nm.^116^

### Metabolic Markers

Several categories of metabolites, including amino acids and lipids, have been pinpointed by researchers to exhibit significant changes in patients with breast cancer when compared to individuals without the disease. These metabolites have integral roles in biological and metabolic processes.^118−120^

**Figure 8:**
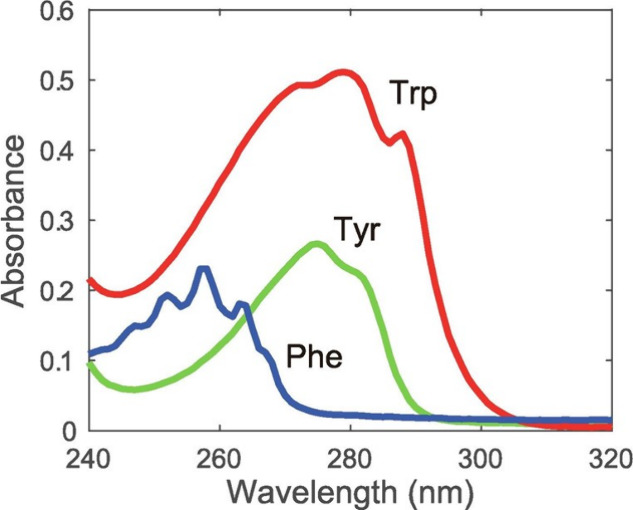
UV–visible absorption of the fluorescent amino acids Phenylalanine, Tryptophan, and Tyrosine. (Reprinted with permission from ref (125). Copyright 2023, Elsevier.)

Amino acids are crucial building blocks for protein synthesis, energy sources, and signaling molecules. However, the dysregulation of amino acid metabolism is a defining characteristic of breast cancer onset, exemplified by the increase in the expression of specific amino acid transporters that influence carcinogenesis; for instance, glutamine metabolism.^121^ In addition, the p53 oncoprotein mutant triggers the production of new glycine/serine in breast cancer tissues and enhances the uptake of essential amino acids, thereby reshaping amino acid metabolism in response to nutrient scarcity and evoking tumor progression.^122^ The absorption peaks of aromatic amino acids; Tyrosine, Tryptophan, and Phenylalanine are around 275 nm, 280 nm and 250–264 nm respectively (Figure 8).^123−126^

Lipids have several contributions toward health and diseases. Specifically, in breast cancer, altered lipids metabolism can lead to enhanced lipogenesis, modified lipid composition and distribution, which supports tumor advancement, invasion, and survival.^127−132^ Unique lipid species, like phosphatidylinositols (PIs), are exclusively identified only in active breast carcinoma with notable variations in lipid ratios between normal and cancerous tissues.^133^ Also, lipid profiles in tumorigenic plasma exhibit specific patterns, such as increased very-low-density lipoprotein (VLDL) subtraction in HER2 positive breast cancer patients.^134^ Studies have demonstrated lipid’s absorption peaks at near-infrared (NIR) region, i.e., 1000–1350 and 1550–1870 nm (Figure 7).^135−137^

Therefore, analyzing these components with their specific signals in breast cancer tissues using photoacoustic spectroscopy allows the differentiation of cancerous tissues to normal ones and can serve as a potential biomarker for diagnosis and therapeutic applications of the disease.

## Clinical Applications of Photoacoustic Spectroscopy (PAS) in Breast Cancer

**Figure 9:**
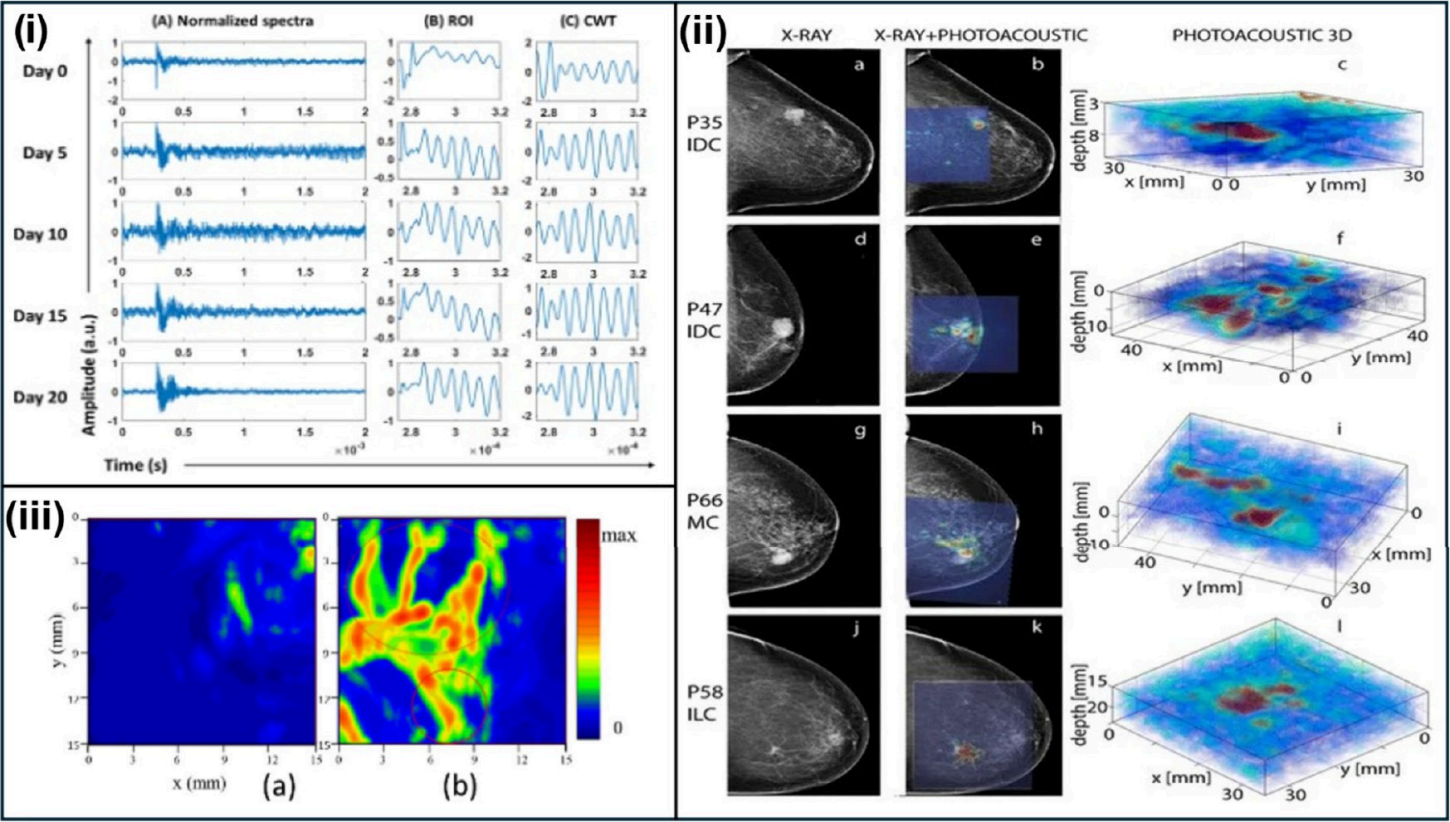
(i) Preprocessed photoacoustic spectra in the region 0 to 2 ms that were recorded *in vivo* at various time points (Day 0, 5, 10, 15, and 20) post-tumor induction in athymic nude mice (A), the corresponding region of interest (ROI) selected spectra in the 0.275–0.32 ms range (B), and continuous wavelet-transformed of the ROI spectra (C). (Reprinted from ref (139). Copyright 2024 American Chemical Society.) (ii) Photoacoustic images overlaid on X-ray mammograms reveal lesions detected in both modalities. 3D reconstructed photoacoustic volumes for Infiltrating Ductal Carcinoma (IDC) in 79-year-old (A–C) and 69-year-old patients (D–F), Mucinous Carcinoma (MC) in an 83-year-old patient (G–I), and Infiltrating Lobular Carcinoma (ILC) in a 65-year-old patient (J–L). Lesions are colocalized on photoacoustic images with X-ray mammograms, offering clear visualization at depths exceeding 20 mm with excellent contrast. (Reprinted from ref (140) Available under Creative Common CC BY license, Copyright 2020 The Authors.) (iii) Reconstructed photoacoustic (PA) images depicting tumor angiogenesis in the XY plane (top view of the tissue). (Reprinted with permission from ref (141). Available under Creative Common CC BY license, Copyright 2009 The Authors.)

Photoacoustic technology is a novel hybrid technique that involves exciting the tissue using laser light and measuring the resulting optically induced ultrasound signals. There is growing interest in the clinical community regarding this new technique and its potential healthcare applications. PAS is renowned for its prowess in high-resolution breast tumor imaging together with remarkable optical contrast.^138^ Research findings have unveiled the use of PA spectroscopy in effectively assessing the progression of breast tumors.^139^ Rodrigues *et al.* have shown the assessment of tumor progression through the analysis of PA spectra over 20 days post-tumor induction in athymic nude mice. Photoacoustic spectra were recorded at different time points (Day 0, 5, 10, 15, and 20) *in vivo* during the progression of breast tumors. The recorded raw, time domain PA spectra (0–2 ms) were subjected to preprocessing, Region of Interest (ROI) selection (0.275–0.32 ms) showing maximum variation with respect to control, and Continuous Wavelet Transformation (CWT) of the ROI spectra for further analysis (Figure 9i). The resulting data was then used to train and test a machine learning model for classifying PA spectra belonging to different time points of tumor progression.^139^ These findings emphasize the potential of PA signal-based classification of breast tumor progression in a preclinical model.

PAS also provides a noninvasive facility for evaluating tumor vascularization in real-time as seen in an *in vivo* investigation that stated PAS revealed a dispersed pattern of vascularization present in the ductal carcinoma while ultrasound image only unveiled the structure.^141^ An *ex vivo*study on a 10 mm (about 0.39 in) spherical human breast cancer sample depicted the capability of three-dimensional (3-D) photoacoustic imaging to view tumors amidst normal biological tissue with outstanding spatial resolution, swift acquisition speed, and an unobtrusive detection approach, ensuring a pain-free diagnosis process.^142^ The spatial resolution of PAS is crucial for detecting tumor-associated changes (TACS) in breast cancer tissues, and this resolution can vary significantly depending on the specific study and methodology employed. For instance, Dahal *et al.* report achieving a near cellular level resolution of 50 μm using an innovative multiphoton excited photoacoustic technique.^143^ In contrast, Liang-Zhong *et al.* detail a photoacoustic computed tomography (PCT) system with a spatial resolution of 0.2 mm and a slice resolution of 1.5 mm along the *Z*-axis.^144^ Additionally, Lu and Mao highlight that the resolution is highly dependent on system-specific parameters, emphasizing the variability and customization of these techniques for different research applications.^145^

PAS can meticulously highlight the spatial distribution of key molecules like collagen, elastin, lipids, melanin, water, and many others within breast tissues, empowering clinicians to gain an unparalleled understanding of the structural alterations in elastin deposition, collagen density, angiogenesis, and other biomolecule metabolism accompanying tumorigenesis.^146^ For instance, recent studies have suggested that the amount and structural alteration of collagen in the ECM are strongly linked to the growth and metastasis of cancer cells. Therefore, collagen can be used as a biomarker to predict the prognosis of breast cancer.^25,41^ Consequently, monitoring hemoglobin photoacoustic signals and tumor oxygenation levels can be used by clinicians to examine angiogenesis in breast cancer (Figure 9iii).^110,115^ This in-depth detailing facility provides great potential in early lesion detection, cancer subtype discrimination, and enhanced precision of any surgical planning by outlining tumor margins with utmost accuracy.^140,147^ Furthermore, PAS holds promise as a critical instrument for tracking treatment efficacy and disease advancement in breast cancer patients as it generates ultrasonographic images that offer functional and molecular insights crucial for breast tumor characterization.^146^ An article displayed the use of PAS beneficial for monitoring the impacts of neoadjuvant therapy in individuals with breast cancer, as it can assess lesions and approximate the likelihood of cancer presence.^148^ Moreover, the integration of photoacoustic spectroscopy with other modalities such as fluorescence imaging, CT scan, X-ray, MRI, and deep learning has been proposed to provide improved resolution, deep penetration, and enhanced specificity and sensitivity for detection, monitoring progression, and treatment of breast cancer (Figure 9i).^149−151^

## Limitations

The complex and dynamic collagen architecture within breast tumors poses difficulties in precisely assessing its density, alignment, and organization through photoacoustic spectroscopy, whereas various other elements of the tumor microenvironment, including blood vessels, immune cells, and ECM proteins, can produce photoacoustic signals that may interfere with collagen signals, complicating the interpretation of generated data. The absence of standardized protocols for carrying out this work and analyzing its data results in inconsistencies among studies and impedes comparisons between them, thereby complicating cross-study evaluations.

A limitation of the current research is the need to elucidate the distinctive roles of other collagen subtypes, such as Collagen types II, VII, IX, X, XIV, XVI, XXI, XXIII, XXIV, XXV, XXVI, XXVII, and XXVIII, in breast cancer. Further studies should also focus on identifying and analyzing variations in photoacoustic (PA) signals generated by these different collagen types to improve our understanding of their contributions to tumor biology and potential as diagnostic markers. Additionally, there is currently no available information about the photoacoustic spectra exclusively for Glycosaminoglycans (GAGs). Furthermore, there is a lack of studies that have demonstrated the absorption properties of pure MMPs or confirmed the presence of PA signals in them within the breast tumor microenvironment.

Despite favorable preclinical outcomes, the clinical translation of photoacoustic spectroscopy for breast cancer detection/diagnosis and management faces serious challenges such as regulatory approval, affordability, and incorporation into available clinical workflows. The ethical concerns regarding the clinical application of photoacoustic spectroscopy, including patient privacy, informed consent, and so forth, must be carefully addressed in both research and clinical settings.

## Conclusion and Outlook

The study of collagen and various other components in the tumor microenvironment, combined with photoacoustic spectroscopy, has provided valuable insights into breast cancer biology. It has demonstrated the impact of these vital components on the carcinogenic process and how photoacoustic spectroscopy can help visualize the internal areas of tumors without surgery or any other invasive techniques. However, further research needs to elucidate the distinctive roles of other collagen subtypes such as Collagen types II, VII, IX, X, XIV, XVI, XXI, XXIII, XXIV, XXV, XXVI, XXVII, and XXVIII in breast cancer, identify and analyze variations in PA signals generated by different collagen types, explore strategies to target them for therapeutic interventions, and develop standardized methods to quantify collagen alterations in breast tumors using photoacoustic spectroscopy, thus allowing reliable comparisons between studies.

Collagen is present in various structural forms, such as fibrillar and network. These forms may have distinct spectral properties, and it is currently unknown if different types of collagens will produce unique PA signals. To overcome this, polarization-sensitive photoacoustic spectroscopy can be utilized to differentiate collagen structures by leveraging their unique optical anisotropies.^152−154^ The similarity of collagen’s spectral features to those of other biological molecules often results in overlapping signals, complicating the analysis. Employing advanced deconvolution techniques and machine learning algorithms can help separate these overlapping features and accurately identify collagen signals. Additionally, selective staining or tagging of collagen with specific contrast agents can enhance its signal relative to other biomolecules.^155−157^ PAS may sometimes show limited sensitivity to collagen’s specific molecular signals, making it hard to distinguish collagen from other extracellular matrix components. Enhancing the signal-to-noise ratio through advanced signal-processing algorithms may improve the collagen-specific absorbers-based diagnostic applications. The lack of comprehensive spectral databases for different collagen types hampers the accurate identification and comparison. Developing extensive, standardized PA spectral libraries for various collagen types and their modifications and integrating these databases into PAS analysis software can address this limitation. Appropriate quantification of collagen concentration using PAS is complex due to the nonlinear nature of photoacoustic signal generation. Techniques like quantitative photoacoustic tomography (QPAT) can provide more accurate measurements of collagen concentration in tissues.^158^ Integrating PAS with other imaging modalities such as MRI, CT, or ultrasound can also help overcome the limitations, offering a more comprehensive approach to collagen analysis.

Additional studies are necessary to confirm the clinical effectiveness of photoacoustic spectroscopy for the detection, prediction, and monitoring of outcomes for breast tumors through extensive clinical trials. There is also a high requirement to advance the development of targeted therapies aimed at specifically disrupting the collagen remodeling process in breast tumors with the potential to inhibit tumor progression and metastasis. With continued research and technological advancements, current limitations can be surpassed, and the capabilities of photoacoustic spectroscopy can be fully harnessed for multiple applications in the field of cancer.

## Vocabulary

GLOBOCAN: Global Cancer Observatory is a project by the International Agency for Research on Cancer (IARC) that offers estimates of cancer incidence, mortality, and prevalence worldwide
Tumor ECM: Tumor extracellular matrix (ECM) is a complex network of proteins and molecules surrounding cancer cells, influencing tumor growth, progression, and metastasis
Fibril: A fibril is a small, thread-like fiber found in biological tissues, formed by the aggregation of proteins or other molecules
Mapping: Identifying and characterizing the spatial distribution of molecular and cellular components within the cells to understand their structure and function
Photoacoustic spectroscopy: Photoacoustic spectroscopy is an analytical technique to measure absorption of light by materials
Photoacoustic signal: Photon induced acoustic signal
PZT: PZT detector is a device that uses lead zirconate titanate (PZT) material to convert mechanical vibrations, such as acoustic waves, into electrical signals
